# Inhibition of metal-induced amyloid β-peptide aggregation by a blood–brain barrier permeable silica–cyclen nanochelator

**DOI:** 10.1039/c9ra02358e

**Published:** 2019-05-08

**Authors:** Jinzhuan Wang, Kun Wang, Zhenzhu Zhu, Yafeng He, Changli Zhang, Zijian Guo, Xiaoyong Wang

**Affiliations:** State Key Laboratory of Coordination Chemistry, School of Chemistry and Chemical Engineering, Nanjing University Nanjing 210023 P. R. China zguo@nju.edu.cn; Nanjing Institute of Product Quality Inspection Nanjing 210028 P. R. China; State Key Laboratory of Pharmaceutical Biotechnology, School of Life Sciences, Nanjing University Nanjing 210023 P. R. China zzz070116@126.com boxwxy@nju.edu.cn; School of Biochemical and Environmental Engineering, Nanjing Xiaozhuang University Nanjing 210017 P. R. China

## Abstract

Alzheimer's disease (AD) is a neurodegenerative malady associated with amyloid β-peptide (Aβ) aggregation in the brain. Metal ions play important roles in Aβ aggregation and neurotoxicity. Metal chelators are potential therapeutic agents for AD because they could sequester metal ions from the Aβ aggregates and reverse the aggregation. The blood–brain barrier (BBB) is a major obstacle for drug delivery to AD patients. Herein, a nanoscale silica–cyclen composite combining cyclen as the metal chelator and silica nanoparticles as a carrier was reported. Silica–cyclen was characterized by scanning electron microscopy (SEM), transmission electron microscopy (TEM), Fourier transform infrared (FT-IR) and dynamic light scattering (DLS). The inhibitory effect of the silica–cyclen nanochelator on Zn^2+^- or Cu^2+^-induced Aβ aggregation was investigated by using a BCA protein assay and TEM. Similar to cyclen, silica–cyclen can effectively inhibit the Aβ aggregation and reduce the generation of reactive oxygen species induced by the Cu–Aβ_40_ complex, thereby lessening the metal-induced Aβ toxicity against PC12 cells. *In vivo* studies indicate that the silica–cyclen nanochelator can cross the BBB, which may provide inspiration for the construction of novel Aβ inhibitors.

## Abstract 2

A BBB-passable nanoscale silica–cyclen chelator effectively reduces the metal-induced Aβ aggregates and related ROS, thereby decreasing the neurotoxicity of Aβ.

## Graphical abstract

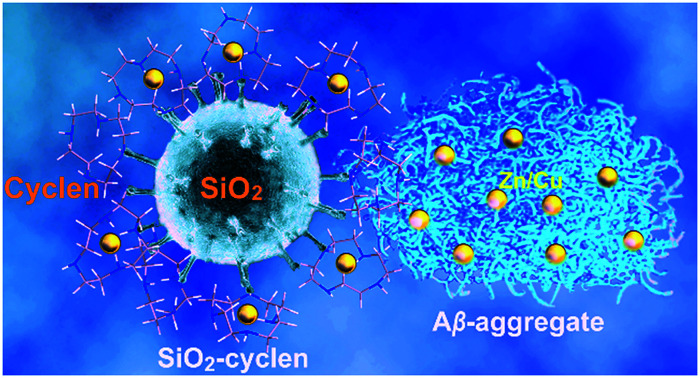

## Introduction

Alzheimer's disease (AD) is a progressive neurodegenerative disorder affecting the memory and cognitive functions of the brain.^1^ Although the molecular mechanism of AD pathogenesis is not clearly understood, much research has demonstrated that polymerization of amyloid β-peptides (Aβ) into amyloid fibrils is a critical step in the pathogenesis.^2^ The pathological hallmark of AD is the aggregation of Aβ, predominantly Aβ_40_ and Aβ_42_ generated from the amyloid precursor protein (APP), which lead to the formation of oligomers and neuritic plaques in the brain.^3,4^ Metal ions, such as Zn^2+^, Cu^2+^ and Fe^3+^, play important roles in the Aβ aggregation and neurotoxicity, because they can readily induce Aβ nucleation and facilitate the formation of neurotoxic reactive oxygen species (ROS).^5^ Thus, metal chelation therapy has been extensively studied as a treatment for AD, which can block the formation of ROS and reduce the Aβ aggregation induced by metal ions.^6^

Although much research has been directed to the development of AD therapy,^7–10^ effective treatments are still unavailable. One of the major reasons is that most of the drug candidates are unable to cross the blood–brain barrier (BBB),^11,12^ which is formed primarily by endothelial cells that line the cerebral microvasculature and surrounding perivascular elements.^13^ Adjacent endothelial cells form complex tight junctions, creating a physical barrier which severely limits the paracellular transport across the BBB.^14^ The BBB allows for the passive diffusion of small lipophilic molecules, whereas limits the passive permeation of hydrophilic substances or molecules with high molecular weight.^15^ Since only lipophilic drugs with a molecular weight less than 450 Da can cross the BBB, most of the traditional drug candidates do not meet this requirement.^16^

In an attempt to overcome the above limitations, nanocarriers have been investigated as drug delivery vehicles to the central nervous system (CNS).^17–20^ The mesoporous silica (SiO_2_) nanoparticles can be utilized to carry various drugs and other functional agents due to their unique properties such as large surface area, stable aqueous dispersion, none toxicity, easy surface modification, excellent biocompatibility and *in vivo* biodegradability.^21–23^ The organically modified SiO_2_ nanoparticles have been used as efficient non-viral vectors to delivery gene therapeutic agent into the CNS *in vivo*.^24^ We and other researchers ever reported that macrocyclic chelator 1,4,7,10-tetraazacyclododecane (cyclen) could reduce the metal-induced Aβ aggregation and neurotoxicity.^25,26^ However, the hydrophilic cyclen (water solubility: 999 g L^−1^) may be hard to cross the BBB.

**Scheme 1:**
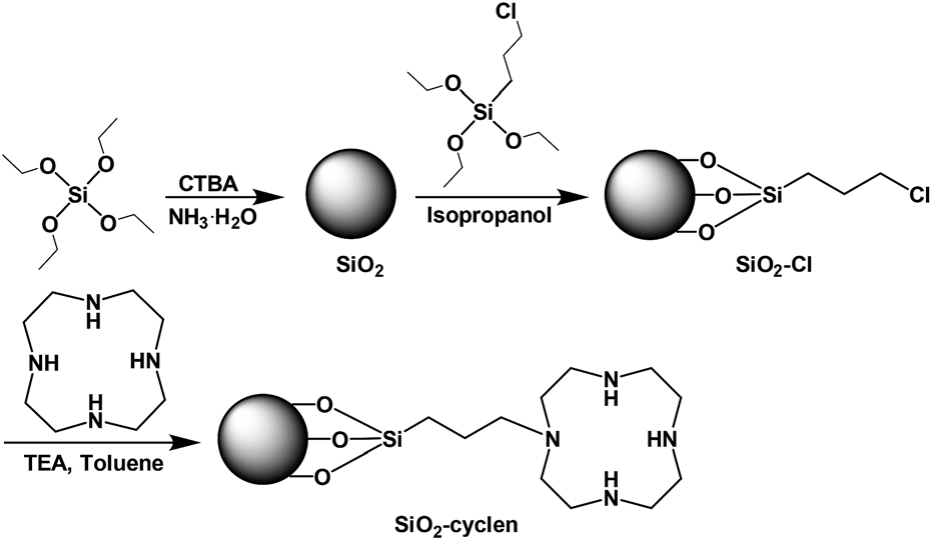
Fabrication route to the SiO_2_–cyclen nanochelator.

In this study, we designed a novel nanoscale chelator SiO_2_–cyclen, which conjugated SiO_2_ nanoparticles as delivery carriers with cyclen as a metal chelator, for inhibiting the metal-induced Aβ toxicity (Scheme 1). The effect of SiO_2_–cyclen nanochelator on Aβ aggregation and neurotoxicity, as well as its BBB permeability were investigated *in vitro* and *in vivo*.

## Results and discussion

### Synthesis and characterization of SiO_2_–cyclen

**Fig. 1:**
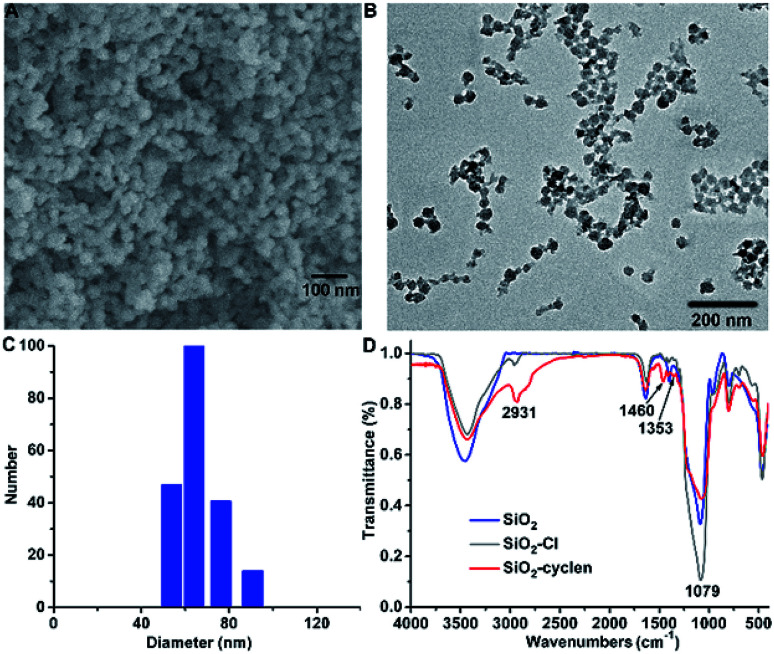
SEM image (A), TEM image (B), DLS size distribution (C) of SiO_2_–cyclen nanochelator, and FT-IR spectra (KBr) of SiO_2_, SiO_2_–Cl and SiO_2_–cyclen (D).

SiO_2_–cyclen nanochelator was designed and fabricated according to a modified literature method.^27^ Cetyltrimethylammonium bromide (CTAB) was used as the cationic surfactant, tetraethylorthosilicate (TEOS) was served as the silica source, and ammonium hydroxide was used as the catalyst. In order to attach the metal chelator cyclen on the surface of SiO_2_ nanoparticles, 3-chloropropyltriethoxysilane was used as a linker to fabricate the nanoscale SiO_2_–cyclen chelator. The morphology of the acquired SiO_2_–cyclen nanochelator was characterized by SEM (Fig. 1A) and TEM (Fig. 1B). The particles are spherical in shape and their size was smaller than 100 nm, which may enter the cells readily under fluid flow conditions.^28^ The average hydrodynamic diameter of SiO_2_–cyclen particles was determined by dynamic light scattering, which give a mean diameter of 65.2 ± 4.9 nm (DLS, Fig. 1C). The size of particles is just within the dimension range (40–100 nm) of nanoparticles that is not only suitable for drug carriers and cellular uptake,^29^ but also suitable for transporting drugs across the BBB.^30^ In the FT-IR spectra, the peak at 1079 cm^−1^ is attributed to the bond of Si–O, and those at 2931, 1460, 1353 cm^−1^ are attributed to the bonds of C–H, N–H, C–N, respectively (Fig. 1D). The relative intensity of C–H and N–H increases as the functionalization goes deeper; by contrast, that of Si–O fluctuates. The changes manifest that cyclen has been linked to the surface of SiO_2_ nanoparticles. The results indicate that the SiO_2_–cyclen nanochelator exists in single particles and disperses separately in aqueous suspension.

### Chelation with Cu^2+^ or Zn^2+^

Cyclen is a metal chelator and has potential to disaggregate the metal-induced Aβ aggregates as previously reported.^25,26,31^ The chelating ability of SiO_2_–cyclen nanochelator was determined by ICP-MS after incubation with Cu^2+^ and Zn^2+^. The Cu and Zn amounts after reacting with SiO_2_–cyclen were 22.18 ± 0.33 and 22.48 ± 0.29 μg mg^−1^ in terms of SiO_2_–cyclen weight, respectively. As a control, no Cu and Zn was detected in SiO_2_–Cl. The results indicate that cyclen tethered to the surface of SiO_2_ nanoparticles still retains the chelating ability to Cu^2+^ and Zn^2+^.

### BCA protein assay

**Fig. 2:**
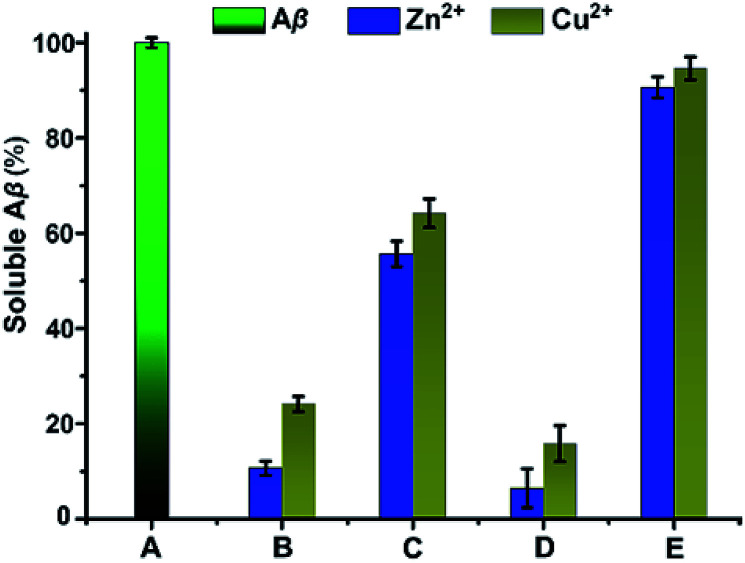
Percentage of soluble Aβ in the solution containing Zn^2+^ or Cu^2+^ with or without SiO_2_–cyclen after incubation at 37 °C for 24 h. (A) Aβ; (B) Aβ + M^2+^; (C) Aβ + M^2+^ + SiO_2_–cyclen; (D) Aβ + M^2+^ + SiO_2_–Cl; (E) Aβ + M^2+^ + cyclen. M^2+^ = Cu^2+^ or Zn^2+^, pH = 7.4, [Aβ_40_] = 40 μM, [Aβ_40_] : [M^2+^] : [chelator] = 1 : 2 : 2.

The effect of SiO_2_–cyclen nanochelator on the Zn^2+^- or Cu^2+^-induced Aβ aggregation was investigated by measuring the percentage of soluble Aβ in the supernatant of the reaction mixtures, with SiO_2_–Cl and cyclen as the references. As shown in Fig. 2, Aβ_40_ is almost completely soluble in the absence of metal ions and chelators. However, soluble Aβ in the supernatant of Aβ reaction mixtures containing Zn^2+^ or Cu^2+^ decreases to 10% and 24%, respectively, indicating that most Aβ is aggregated and deposited by metal ions. In the presence of SiO_2_–cyclen, the solubility of Aβ increases obviously, suggesting that the SiO_2_–cyclen nanochelator can inhibit the metal-induced aggregation of Aβ. As a comparison, SiO_2_–Cl can hardly inhibit the metal-induced Aβ aggregation. These results show that the cyclen-modified mesoporous silica nanoparticles still have metal-chelating function and can inhibit the metal-induced Aβ aggregation.

### Inhibition of ROS generation

**Fig. 3:**
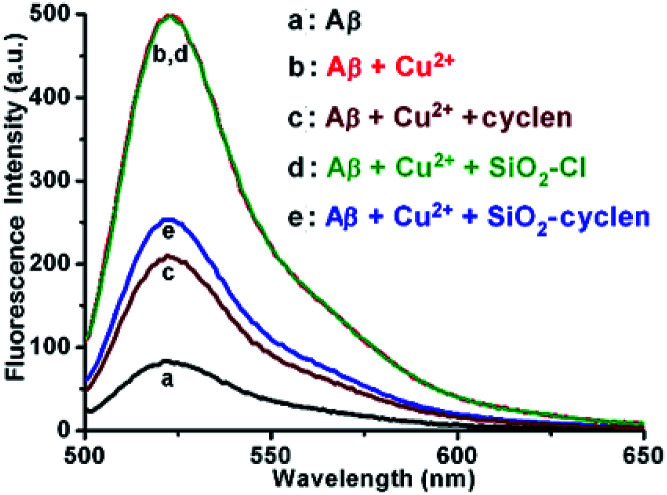
Fluorescence of DCF (*λ*_ex_ = 485 nm) reflecting the effect of SiO_2_–cyclen on the production of H_2_O_2_ by the Cu–Aβ_40_ complex. [Aβ_40_] = 0.8 μM, [Cu^2+^] = [chelator] = 0.6 μM, [HRP] = 0.04 μM, [DCFH] = 100 μM, [ascorbate] = 10 μM, pH = 7.4.

Redox-active metal ions are crucial for the production of ROS and oxidative stress. Aβ could promote the production of ROS in the presence of redox-active metal ions, leading to pathological oxidative stress in AD.^32^ Chelating agents can reduce the generation of ROS through removing Cu^2+^ ions from the Cu–Aβ complex. The generation of ROS induced by the Cu–Aβ complex was monitored using 2′,7′-dichlorofluorescein diacetate (DCFH-DA). DCF is a fluorescent marker derived from the reaction of non-fluorescent DCFH with ROS in the presence of horseradish peroxidase (HRP).^33^ The fluorescence intensity of DCF correlates with the amount of reactive oxygen radicals. As shown in Fig. 3, strong fluorescence of DCF is measured at 522 nm for the Cu–Aβ_40_ system without the SiO_2_–cyclen nanochelator (b); in the presence of SiO_2_–cyclen, the fluorescence intensity decreases obviously (e). The results indicate that SiO_2_–cyclen can reduce the generation of ROS induced by the Cu–Aβ_40_ complex. In contrast, SiO_2_–Cl shows no effect on the reduction of ROS (d), because it does not coordinate with the Aβ-bound Cu^2+^ and hence can hardly influence the Cu–Aβ_40_-mediated redox chemistry. These results indicate that the SiO_2_–cyclen nanochelator reduces the production of ROS induced by Cu–Aβ complex almost as effectively as cyclen.

### Morphology changes of Aβ aggregates

**Fig. 4:**
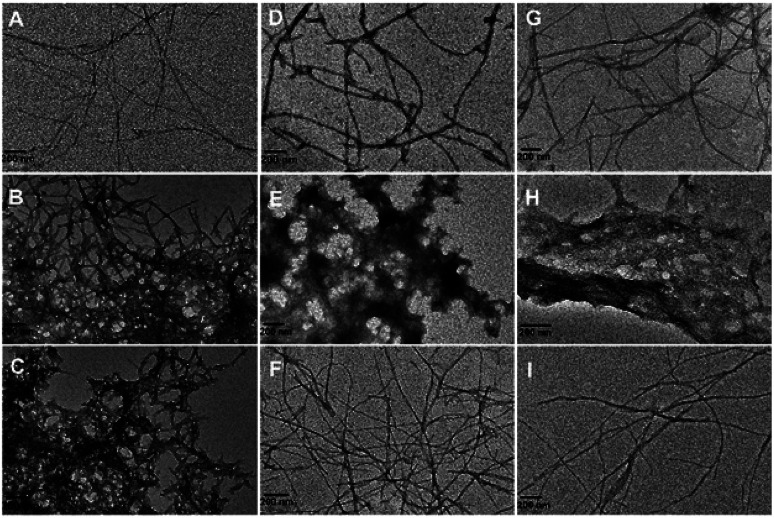
TEM images of Aβ (A), Aβ + Cu^2+^ (B), Aβ + Zn^2+^ (C), Aβ + Cu^2+^ + SiO_2_–cyclen (D), Aβ + Cu^2+^ + SiO_2_–Cl (E), Aβ + Cu^2+^ + cyclen (F), Aβ + Zn^2+^ + SiO_2_–cyclen (G), Aβ + Zn^2+^ + SiO_2_–Cl (H), Aβ + Zn^2+^ + cyclen (I), respectively, after incubation at 37 °C for 24 h. pH = 7.4, [Aβ_40_] = 20 μM, [Aβ_40_] : [M^2+^] : [chelator] = 1 : 2 : 2.

Negative staining TEM was exploited to investigate the effect of the SiO_2_–cyclen nanochelator on the morphology of metal-induced Aβ aggregates. The images of Zn^2+^- or Cu^2+^-induced Aβ aggregates in the absence and presence of the nanochelator are shown in Fig. 4. Only long unbranched fibrils, a typical structure for amyloid fibrils, were observed in the solution of Aβ_40_ (Fig. 4A). However, after Zn^2+^ or Cu^2+^ was added, large amounts of amorphous aggregates were formed in the solution of Aβ_40_ (Fig. 4B and C), which are consistent with our previous observations.^34,35^ In the presence of SiO_2_–cyclen, the metal-induced Aβ_40_ aggregates were almost disappeared, and the morphology of the samples was similar to that of Aβ_40_ samples (Fig. 4D and G). Cyclen also inhibited the Zn^2+^- or Cu^2+^-induced Aβ_40_ aggregation and made the morphology similar to that of Aβ_40_ alone (Fig. 4F and I). More aggregates were observed in the presence of SiO_2_–Cl owing to its inability to chelate Zn^2+^ or Cu^2+^ (Fig. 4E and H). The results indicate that the SiO_2_–cyclen nanochelator can inhibit the Zn^2+^- or Cu^2+^-induced Aβ_40_ aggregation.

### Inhibition of neurotoxicity

**Fig. 5:**
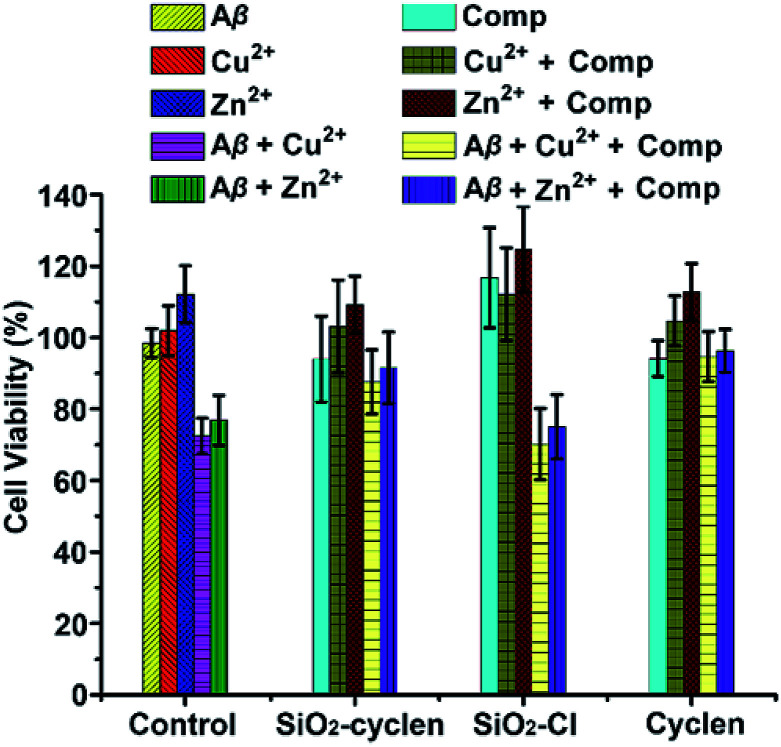
Neurotoxicity of Aβ_40_ in the absence or presence of metal ions and chelators against PC12 cells after incubation at 37 °C for 48 h. [Aβ_40_] = 10 μM; [Aβ_40_] : [M^2+^] : [chelator] = 1 : 2 : 2.

The neurotoxicity of Zn^2+^– or Cu^2+^–Aβ_40_ complexes against PC12 cells was investigated by the MTT assay. The inhibition of SiO_2_–cyclen nanochelator against the Aβ_40_-induced neurotoxicity was shown in Fig. 5, with cyclen and SiO_2_–Cl as the references. Aβ_40_ in the presence of Zn^2+^ or Cu^2+^ was quite toxic to the rat pheochromocytoma PC12 cells (cell viability is about 70%), while Zn^2+^, Cu^2+^, and Aβ_40_ alone are almost nontoxic. In the presence of SiO_2_–cyclen, the cell viability in the Zn^2+^–Aβ_40_ system increased from 74% to 91%, and that in the Cu^2+^–Aβ_40_ system increased from 71% to 93%, respectively. Interestingly, SiO_2_–cyclen and its Zn^2+^ or Cu^2+^ complex is nontoxic toward the cells. In the presence of SiO_2_–Cl, the cell viability is similar to that in the presence of Aβ_40_ and Zn^2+^ or Cu^2+^, indicating that SiO_2_–Cl had no effect on the neurotoxicity of the Zn^2+^– or Cu^2+^–Aβ_40_ complex. After incubating with cyclen, the cell viability was above 90%, even in the present of Zn^2+^– or Cu^2+^–Aβ_40_ complex. These results demonstrate that the SiO_2_–cyclen nanochelator can inhibit the neurotoxicity of Zn^2+^– or Cu^2+^–Aβ_40_ complexes and enhance the viability of neuron cells.

### Blood–brain barrier permeability

To evaluate the *in vivo* BBB permeability of the SiO_2_–cyclen nanochelator, animal experiments were carried out by using C57BL/6J mice. The amount of silicon in mice brain pre- or post-injection of SiO_2_–cyclen was listed in Table 1. The amount of Si increased after 6 h, thus suggesting that the nanochelator could penetrate the BBB of mice. The amount of Si decreased at 24 h probably due to the metabolism of SiO_2_–cyclen *in vivo*. The SiO_2_–cyclen nanochelator may cross the BBB *via* the adsorptive or receptor-mediated transportation, which is similar to the situation of insulin, albumin and low density lipoprotein receptor as reported previously.^36^

**Table 1:** The amount of silicon (μg g^−1^) in C57BL/6J mice brain determined by ICP-MS in terms of body weight pre- or post-injection of SiO_2_–cyclen

Pre-injection	Post-injection		
	6 h	12 h	24 h
5.42 ± 0.32	11.11 ± 0.41	13.09 ± 0.37	10.29 ± 0.34

## Experimental

### Materials and methods

Cetyltrimethylammonium bromide (CTAB), ammonium hydroxide, tetraethylorthosilicate (TEOS), isopropanol, 3-chloropropyltriethoxysilane, 1,4,7,10-tetraazacyclododecane (cyclen), trimethylamine (TEA), toluene, and HNO_3_ were purchased from J&K Ltd. (Beijing, China). Human Aβ_40_ was purchased from GL Biochem Ltd. (China). Zinc acetate dehydrate, copper chloride, 2′,7′-dichloro-fluorescin diacetate (DCFH-DA), tris(hydroxymethyl)aminomethane (Tris), horseradish peroxidase (HRP), ascorbate, phosphotungstic acid, poly-l-lysine solution (0.01%), nerve growth factor-7*S*, and 3-(4,5-dimethyl-2-thiazolyl)-2,5-diphenyl-2-*H*-tetrazolium bromide (MTT) were purchased from Sigma-Aldrich. Micro BCA protein assay kit was purchased from Beyotime biotech. Ltd. (China). All the aqueous solutions were prepared using Milli-Q water and filtered through a 0.22 μm filter (Millipore). Stock solutions of Aβ_40_, Cu^2+^, and Zn^2+^ were prepared according to the reported procedures.^31^

The scanning electron microscopy (SEM) images were obtained using a Hitachi S-4800 high resolution SEM on the conductive adhesive tapes. The transmission electron microscopy (TEM) images were obtained using a JEOL JEM-2100 transmission electron microscope at an accelerating voltage of 100 kV. Hydrodynamic diameters were determined using a BI-200SM dynamic light scattering system (DLS, Brookhaven Instruments Co., Holtsville, NY). Fourier transform infrared (FT-IR) spectra (KBr pellets) were recorded on a Bruker VECTOR22 spectrometer in the range of 500–4000 cm^−1^. The content of Si was determined on an inductively coupled plasma mass spectrometer (ICP-MS) using a standard Plasma-Quad II instrument (VG Elemental, Thermo OptekCorp.). Fluorescence spectra were recorded on an LS-50B spectrofluorimeter (Perkin-Elmer, USA). MTT assay and fluorescence spectra (*λ*_ex_ = 485 nm) in the range of 505–650 nm were measured by a Varioskan Flash microplate reader (Thermo Scientific).

### Preparation of SiO_2_–cyclen nanochelator

SiO_2_ nanoparticles were synthesized by a modified literature procedure.^37,38^ CTAB (0.5 g) was dispersed in water (200 mL) with ultrasonic wave. Ammonium hydroxide (0.75 mL, 28 wt% NH_3_ in water) was then added to the solution with strong stirring at room temperature, and TEOS (2.0 mL) was dropped in slowly, giving rise to a white slurry. The resulting product was centrifuged after 3 h, the CTAB was washed out by ethanol and water, and SiO_2_ nanoparticles were obtained after drying at vacuum. SiO_2_–Cl nanoparticles were prepared according to the modified literature procedure.^39^ SiO_2_ nanoparticles (200 mg) were dispersed in isopropanol (200 mL) solution and were allowed to react with 3-chloropropyltriethoxysilane (4.0 mL, in excess) at 100 °C under nitrogen for 24 h. Excess 3-chloropropyltriethoxysilane was removed by centrifugation and redispersion in ethanol and water, followed by drying at room temperature. Cyclen was tethered to the surface of SiO_2_–Cl nanoparticles according to the literature procedure.^40^ In a typical reaction, cyclen (2.0 g, in excess) and triethylamine (6.0 mL) were poured into a flask containing toluene (200 mL) and the SiO_2_–Cl nanoparticles with vigorous stirring under argon atmosphere and reflux for 16 h. The sample was washed with water and ethanol by centrifugation and SiO_2_–cyclen nanochelators were obtained after drying at vacuum. The surface morphology, size, and components of the nanoparticles were investigated by SEM, TEM, DLS, and FT-IR.

### Chelation ability of SiO_2_–cyclen

SiO_2_–cyclen (6.0 mg) and SiO_2_–Cl (6.0 mg) nanoparticles were dispersed into CuCl_2_ (1.0 mol L^−1^, 2.0 mL, in excess) or Zn(Ac)_2_ (1.0 mol L^−1^, 2.0 mL, in excess) solutions respectively, and cultured at 37 °C for 3 h. The samples were cleaned by water to remove the excess CuCl_2_ or Zn(Ac)_2_, and digested by concentrated HNO_3_ at 95 °C for 3 h. The amounts of chelated Cu^2+^ or Zn^2+^ in the SiO_2_–cyclen nanochelators were determined by ICP-MS.

### BCA protein assay

Aβ_40_ (40 μM) in buffer solution (20 mM Tris–HCl/150 mM NaCl, pH 7.4, 197.6 μL) was incubated with or without Zn(Ac)_2_ or CuCl_2_ (80 μM) for 5 min at room temperature. Metal chelator (2.4 μL, 80 μM) was added to the solution and incubated at 37 °C for 24 h. The solution was centrifuged at 12 000 rpm for 30 min and each sample was transferred to individual wells of a flat-bottomed 96-well plate (Corning Costar Corp). The concentration of peptide in the supernatant was analyzed by the Micro BCA protein assay.

### ROS assay

DCFH-DA stock solution (1 mM) as ROS probe was prepared in buffer (20 mM Tris–HCl/150 mM NaCl, pH 7.4). Horseradish peroxidase (HRP) stock solution (4 μM) was prepared with the same buffer. Sample solutions containing Aβ_40_ (0.8 μM) and CuCl_2_ (0.6 μM) were incubated with or without chelators (0.6 μM) at 37 °C for 20 h. Ascorbate (10 μM) was added to each sample and incubated at 37 °C for 1 h. The samples were transferred to individual wells of a flat-bottomed 96-well black plate. HRP (2 μL, 0.04 μM) and DCFH-DA (20 μL, 100 μM) were added to each solution and incubated for 10 min in the dark at room temperature. Fluorescence spectra (*λ*_ex_ = 485 nm) in the range of 505–650 nm were measured by a Varioskan Flash microplate reader (Thermo Scientific).

### Morphology of Aβ aggregates

Aβ_40_ (20 μM) in buffer solution (20 mM Tris–HCl/150 mM NaCl, pH 7.4, 197.6 μL) was incubated with or without Zn(Ac)_2_ or CuCl_2_ (40 μM) for 5 min at room temperature. Metal chelator (2.4 μL, 40 μM) was added to the solution and incubated at 37 °C for 24 h. An aliquot of each solution (5 μL) was spotted onto carbon-coated copper grids for 30 min. The samples were stained with phosphotungstic acid [1.5% (w/v), pH 7.4]. The grids were blotted with filter paper to remove excess solution and air-dried before analysis on the TEM, operating with a voltage of 100 kV.

### Cell viability

PC12 cells (American Type Culture Collection) were cultured in RPMI-1640 medium supplemented with 5% fetal bovine serum (FBS), antibiotics, and 10% horse serum in a 5% CO_2_ humidified environment at 37 °C. The cells were plated at a density of 6000 cells per well on a poly-l-lysine-coated 96-well plates, and differentiated with 100 ng mL^−1^ of nerve growth factor (NGF) in DMEM medium supplemented with 5% FBS at 37 °C with 5% CO_2_ for 48 h. The cytotoxicity of Aβ_40_ and Zn^2+^– or Cu^2+^–Aβ_40_ with or without chelators toward the cells was measured after incubation for 48 h. The cells were treated with MTT (20 μL, 5 mg mL^−1^ in PBS) for 4 h at 37 °C and lysed in DMSO for 30 min at room temperature in the dark. Absorbance values of formazan were determined by a Varioskan Flash microplate reader (Thermo Scientific) at 570 nm. The optical density (OD) was used to calculate the percentage of cell viability relative to the untreated control values, that is, (OD_test_ − OD_blank_)/(OD_control_ − OD_blank_) × 100%, and the mean of three replicates was taken as the final result.

### *In vivo* BBB penetration assay

C57BL/6J mice (8 week, male, 20 g, *n* = 12) were selected as animal models. SiO_2_–cyclen nanochelators were injected intravenously (8 mg kg^−1^ body weight) into the mice (3 mice in each group), and the brains of mice were acquired after 6 h, 12 h and 24 h, with mice without injection as controls. The brain samples were digested by concentrated HNO_3_ at 95 °C, 30% H_2_O_2_ and concentrated HCl at 37 °C. The silicon amount in the samples was determined by ICP-MS. All experimental procedures are in accordance with the Guidelines for Care and Use of Laboratory Animals of Nanjing University, and experiments were approved by the Animal Ethics Committee of the Model Animal Research Center of Nanjing University.

## Conclusions

In this study, a novel nanoscale chelator, SiO_2_–cyclen, was reported, which is composed by cyclen as the metal-chelating unit and silica nanoparticle as a carrier of cyclen, for inhibiting the toxicity of Aβ aggregates. The results show that the SiO_2_–cyclen nanochelator can effectively inhibit Aβ aggregation, reduce the generation of reactive oxygen species induced by the Cu–Aβ_40_ complex, and protect cells from the metal-induced Aβ toxicity. Blood–brain barrier is a dynamic barrier protecting the brain against invading organisms and unwanted substances; it is also the most important barrier impeding the drug transport into the brain *via* the blood circulation.^41^*In vivo* study demonstrated that the SiO_2_–cyclen nanochelator can overcome the drawbacks of small chemicals (>400 Da) or peptides in passing across the BBB, which may has some reference value for the design of novel Aβ inhibitors.

## Conflicts of interest

There are no conflicts to declare.

## Supplementary Material
